# A Case of Pneumatic Rectal Perforation Caused by Compressed Air

**DOI:** 10.7759/cureus.9954

**Published:** 2020-08-23

**Authors:** Rahul Gupta, Pradip Pokharia, Ujjwal Daspal, Houssem Ammar

**Affiliations:** 1 Gastrointestinal Surgery, Synergy Institute of Medical Sciences, Dehradun, IND; 2 Radiology, Synergy Institute of Medical Sciences, Dehradun, IND; 3 Anaesthesiology, Synergy Institute of Medical Sciences, Dehradun, IND; 4 Surgery, Sahloul Hospital, Sousse, TUN

**Keywords:** rectum, intestinal perforation, large colon, surgery, pneumoperitoneum, barotrauma

## Abstract

Rectal perforation is a rare cause of acute abdomen. The most common cause of rectal perforation is trauma. Barotrauma due to the injection of compressed air in the rectum is an extremely rare cause of rectal perforation. We report a case of extensive pneumoperitoneum with abdominal compartment syndrome caused by rectal perforation secondary to the forceful injection of compressed air through the perineum. The patient was successfully managed by immediate relief of abdominal compartment syndrome by needle decompression followed by surgical repair of rectal perforation.

## Introduction

High-pressure compressed air is being increasingly used in many industrial workplaces. A blow gun dust cleaner is a commonly used pneumatic tool in the industries [1]. The improper use of the cleaner can lead to potentially life-threatening pneumatic injuries to the factory workers. The mean resting anal pressure in adults is 0.84 kg/cm^2^ [2]. However, the air pressure through the cleaner is about 10 times higher than the resting anal pressure. Therefore, it can easily overcome the anal sphincter pressure and cause sudden rapid inflation of the colon and rectum. The compressed air can even penetrate barriers, such as clothes. The bursting pressure of the bowel wall is about 0.415 kg/cm^2^ [3]. Hence, the rapid insufflation of the large intestine with compressed air leads to bowel perforation. Following perforation, the patient develops sudden onset abdominal pain, distension and respiratory due to tension pneumoperitoneum [4]. Because of its rarity, the workers are not being made aware that accidental or purposeful use of compressed air close to the perineum can lead to serious colorectal injuries [5,6]. We report a case of severe rectal laceration occurring due to compressed air leading to abdominal compartment syndrome and respiratory distress.

## Case presentation

**Figure 1:**
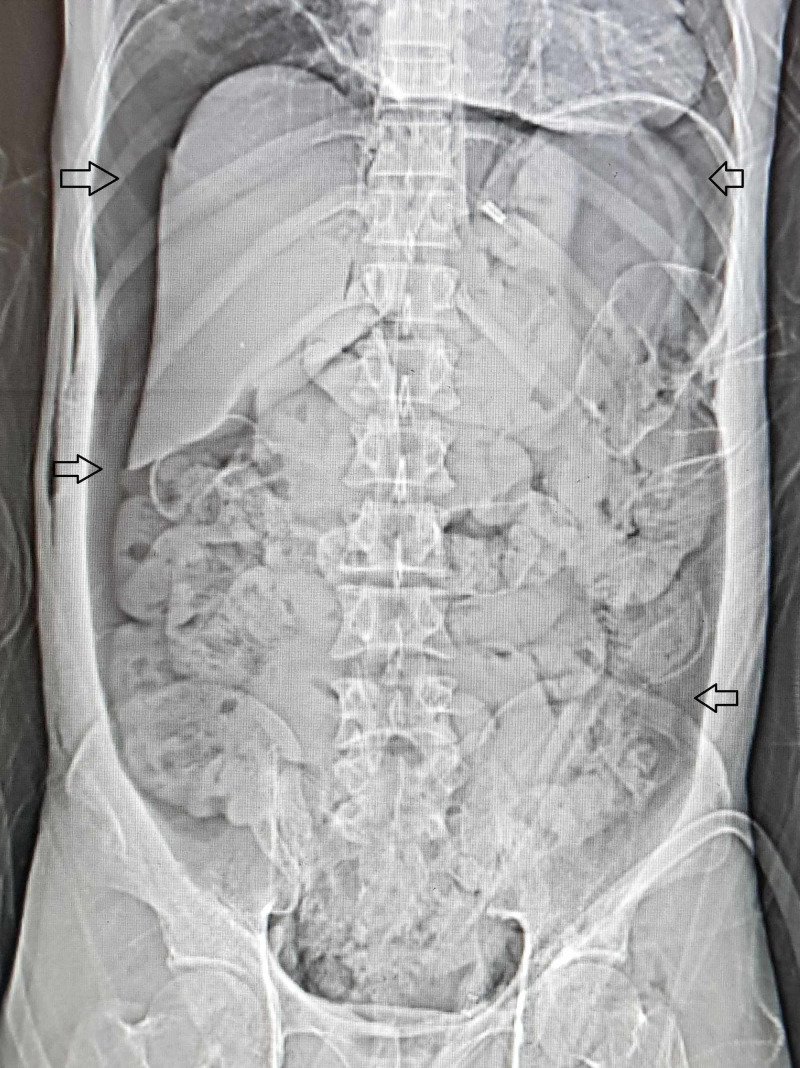
Abdominal radiograph showing the extensive pneumoperitoneum with outlining of the liver, spleen and the small intestine (arrows).

A 40-year-old man presented with sudden onset abdominal pain, distension and per rectal bleeding. He reported that four hours prior, his friend placed air nozzle of compressed air gun near his perineum while they were cleaning out the dust from their body and clothes in a factory. Immediately, after that he developed abdominal distension and pain. The patient had no comorbidities. On clinical examination, the patient was tachypneic and the abdomen was grossly distended, tympanic and tender. On per rectal examination, fresh blood was present in the rectum. No rectal mucosal injury could be appreciated. Abdominal radiograph revealed extensive pneumoperitoneum (Figure 1).

**Figure 2:**
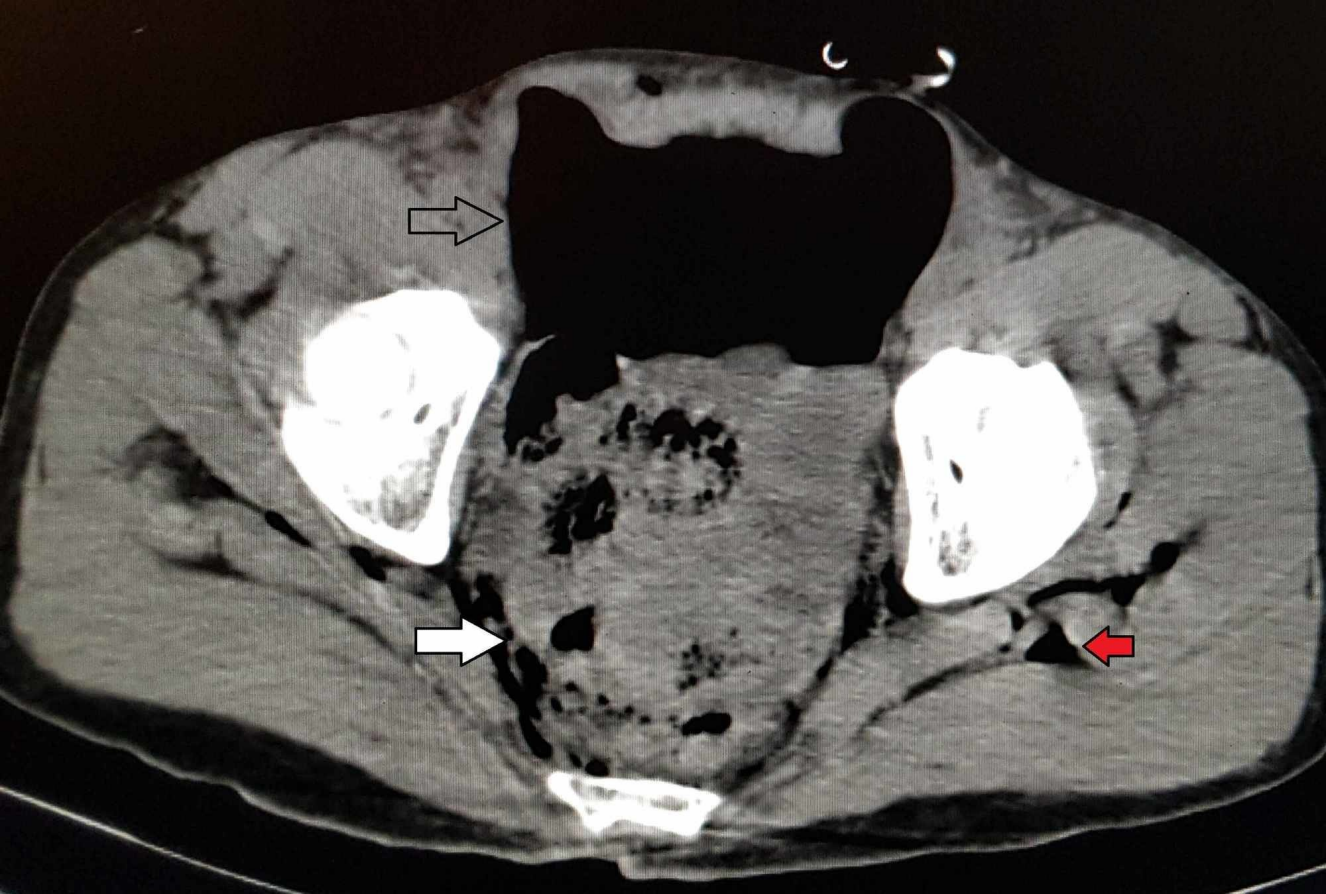
CT of the pelvis showing the pneumoperitoneum (arrow), air in the mesorectum (white arrow) and subcutaneous emphysema (red arrow).

Blood investigations were unremarkable. Contrast-enhanced CT revealed gross pneumoperitoneum, air in the perirectal fat tissue and subcutaneous emphysema suggestive of rectal perforation (Figure 2). 

**Figure 3:**
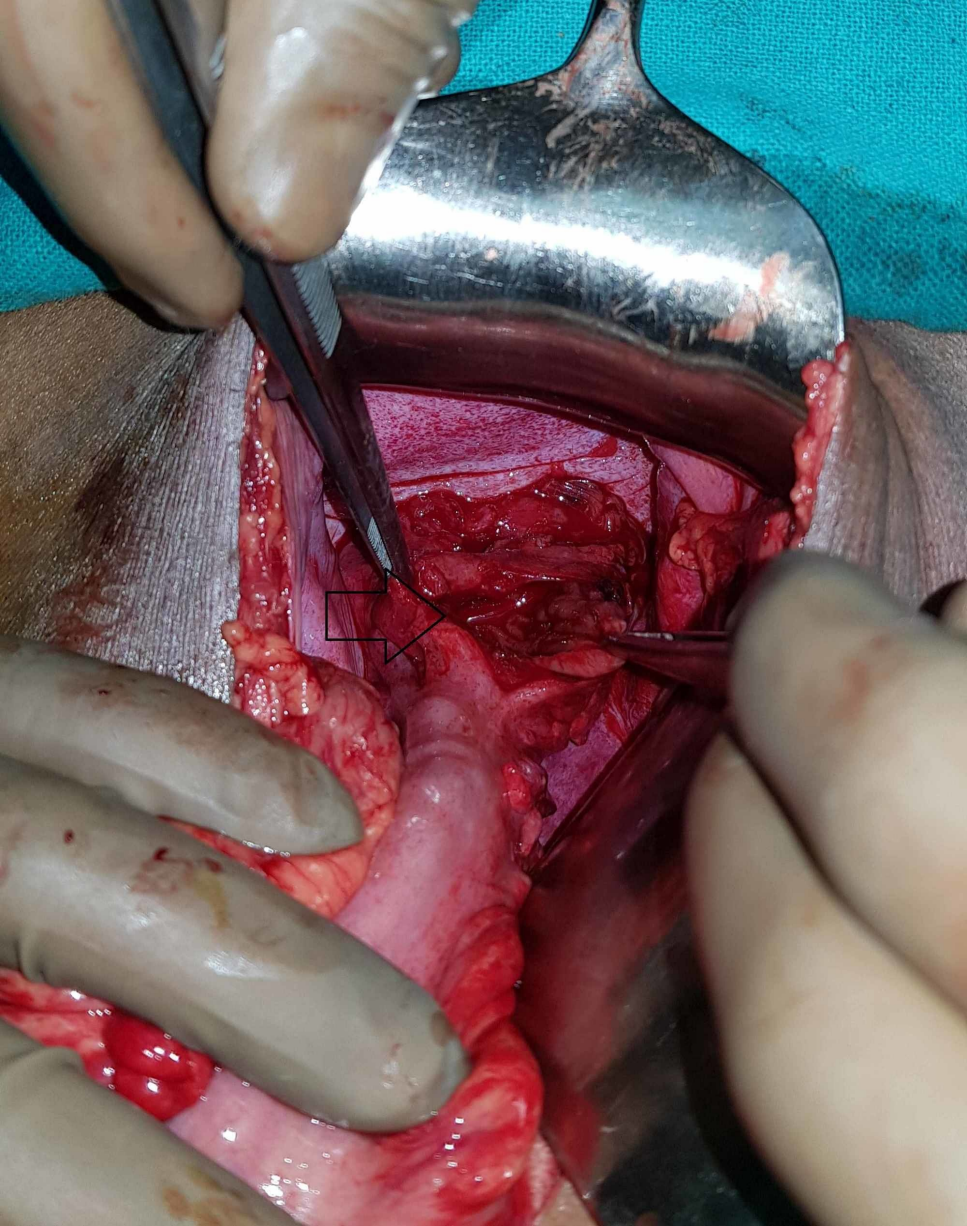
Intraoperative photograph showing the large perforation in the anterior wall of the rectum (arrow).

Urgent abdominal decompression was done using large bore needle to reduce the respiratory distress. On laparotomy, air gushed out of the abdominal cavity and about 100 ml of hemoperitoneum was present. The upper and middle third of the rectum was lacerated with presence of a large 4 x 4 cm full thickness perforation in the anterior wall of the rectum with fecal matter coming out of it (Figure 3). 

On examination through the perforation, no full thickness injury to the posterior rectal wall was found. The perforation was primarily repaired in two layers by 3-0 polydioxanone sutures, and diverting loop sigmoidostomy was performed. The operative time was 120 minutes and blood loss was 100 ml. Postoperative recovery was uneventful with hospital stay of six days. After two months, the patient underwent colostomy closure. At the last follow-up of six months after colostomy closure, he is symptom free. 

## Discussion

The reported incidence of rectal trauma is approximately 1%-3% in civilian trauma centers with the most common cause being gunshot injury [7]. Rectal perforation secondary to barotrauma is rare [5,8]. The high-pressure compressed air used by the industrial workers has been reported to cause such severe rectal pneumatic injuries as seen in the present case [5,6,8]. Colorectal injuries can occur when the nozzle of the air gun is placed close to the anus even when the perineum is covered by the clothes. The highly compressed air acts like a solid body that can forcefully open the anal sphincter. 

The most common sites of injury in such cases are the rectosigmoid junction and sigmoid colon as seen in the current case [5,8]. The colonic injuries vary from simple serosal tears to full-thickness perforations. Also, the injuries can occur at multiple sites. The extent of injuries depends on the resultant intraluminal pressure, the airflow velocity, the duration of exposure to the compressed air, the anal resting pressure and the distensibility of the bowel wall. 

In suspected cases of rectal injury, digital rectal examination and rigid proctoscopy should be performed. These bedside procedures can easily detect injuries in the mid- and lower-third of the rectum. However, for higher injuries, abdominal imaging should be conducted. The large amount of air in the peritoneum can be easily visualized on chest or abdominal radiograph taken in erect position as seen in the index case [4]. In doubtful cases, CT with or without rectal contrast is the most useful non-invasive test to confirm the diagnosis. If the CT is negative, then sigmoidoscopy can be performed in suspected cases. 

Due to the high pressure, large amount of air enters the peritoneum through the perforation leading to severe abdominal distension and respiratory distress [4]. Immediate abdominal decompression by large bore needle helps in relieving the abdominal distension and respiratory distress. The management of rectal injury depends on the location of the perforation [7]. Most of the extraperitoneal rectal perforations can be managed conservatively. Some cases may require presacral drainage and distal rectal wash. In contrast, intraperitoneal rectal perforations should be managed surgically. Small perforations (<25% circumferential involvement) can be repaired primarily [3]. Large defects or lacerations (>25% circumferential involvement) require resection and anastomosis. Fecal diversion may be performed in cases with fecal peritonitis, extensive rectal injuries, hemodynamic instability and those with pelvic sepsis [8].

## Conclusions

Colorectal injuries should be suspected in patients exposed to perineal barotrauma. These patients are at high risk for development of intra-abdominal compartment syndrome. Intraperitoneal pneumatic colorectal perforations should be managed by immediate surgery in order to prevent development of fecal peritonitis, pelvic sepsis and septic shock.
